# Supplementary material for: Seismic evidence for uniform crustal accretion along slow-spreading ridges in the equatorial Atlantic Ocean
Source: Nat Commun. 2022 Dec 17;13:7809. doi: 10.1038/s41467-022-35459-z (PMC9759516; doi:10.1038/s41467-022-35459-z)
Supplement: Supplementary file 1 — Supplementary information [file 41467_2022_35459_MOESM1_ESM.pdf]

Seismic evidence for uniform crustal accretion along slow-spreading ridges in the equatorial Atlantic Ocean

Zhikai Wang[1,*] and Satish C. Singh[1]

*Corresponding author: zwang@ipgp.fr

1. Université Paris Cité, Institut de Physique du Globe de Paris, CNRS, 1 rue Jussieu, Paris 75238, France

Supplementary Information

This PDF file includes:

Supplementary Figures 1 to 15;

Supplementary Tables 1 to 4;

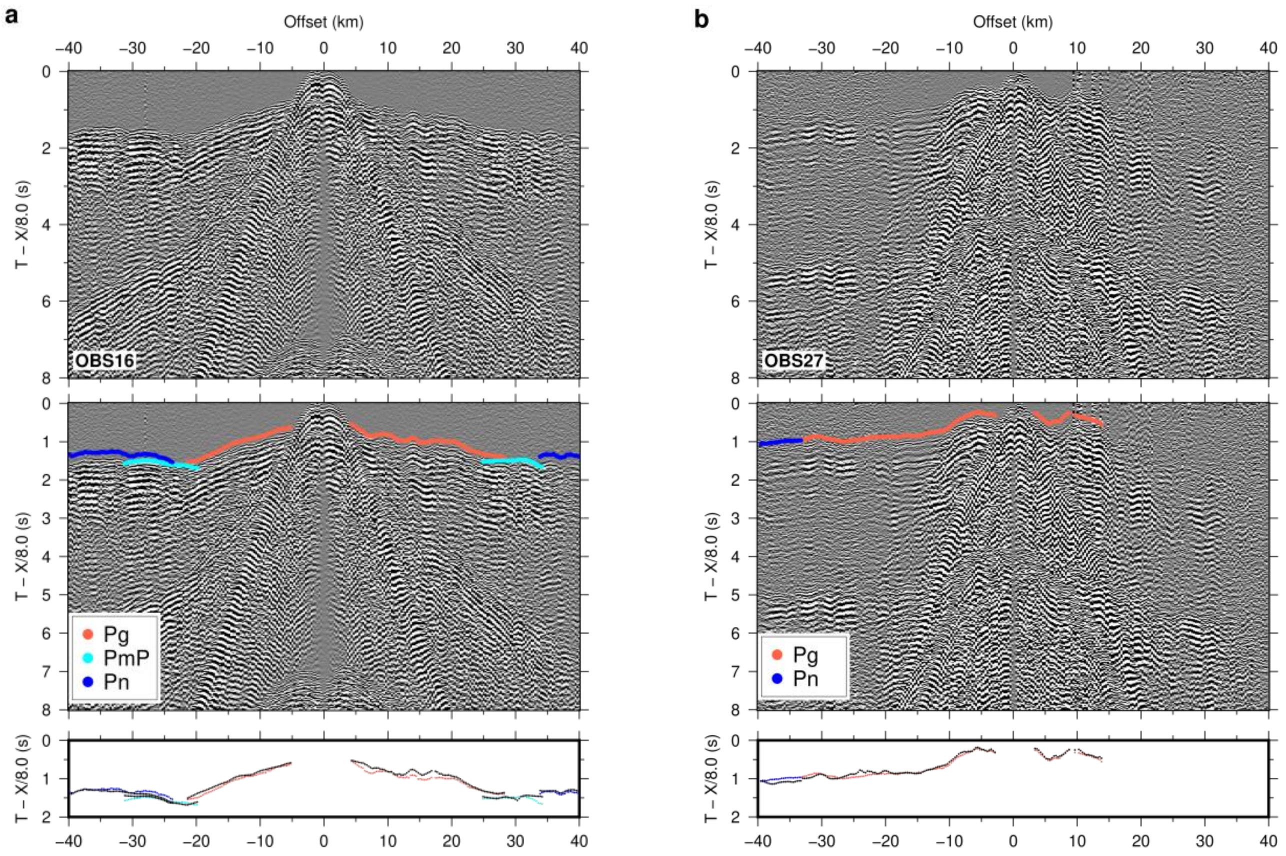

Supplementary Fig. 1. OBS data and P-wave arrivals (Pg, PmP and Pn). **a**, OBS16 and **b**, OBS27. Top: Receiver gathered seismic data after band-pass filtering plotted at a reduced travel time using a reduction velocity of 8.0 km/s. T is the travel time and X is the offset. Middle: Receiver gathered seismic data with the travel time picks superimposed. Pg: Orange, PmP: Cyan, Pn: Blue. Bottom: Comparisons of the picked travel times and modelled travel times (black dots). The effects of bathymetry variation are removed in all plots by subtracting the vertical propagation time in water beneath each source, assuming a water velocity of 1.5 km/s.

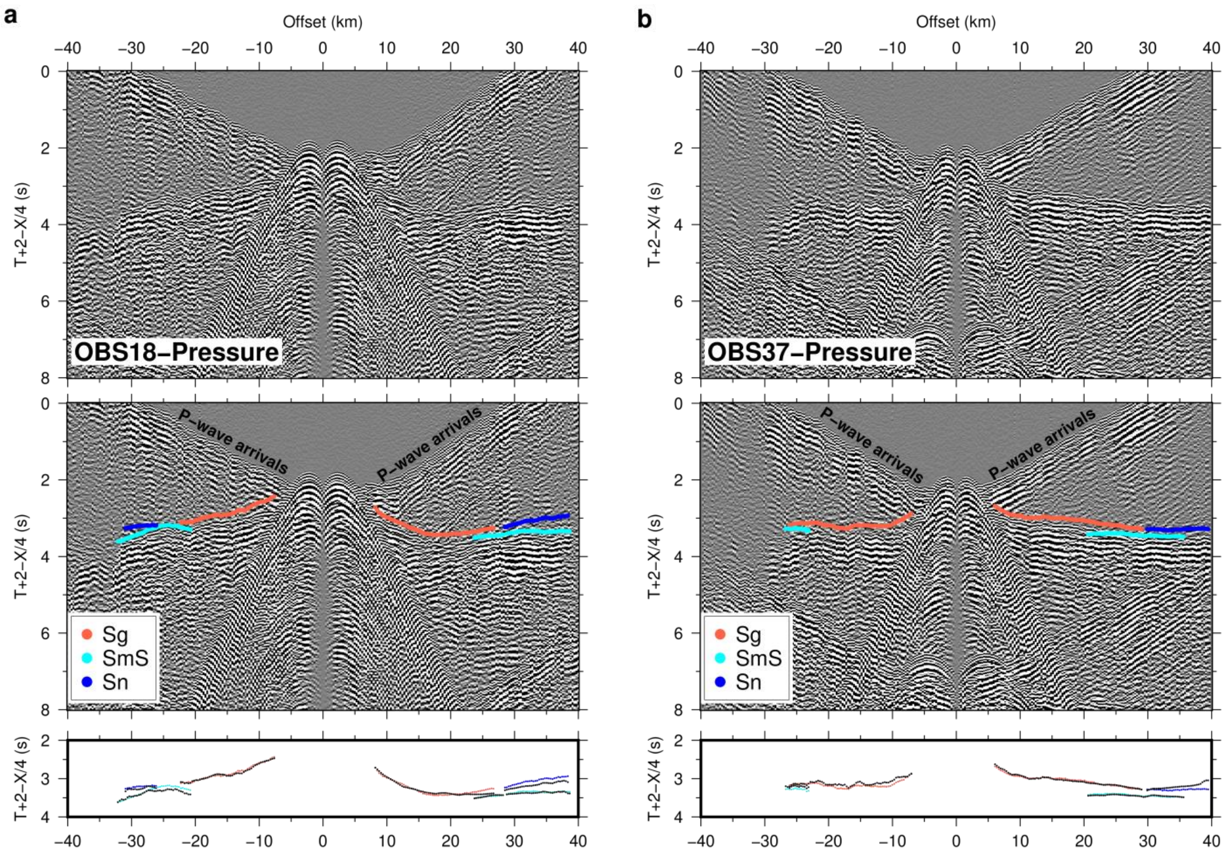

Supplementary Fig. 2. OBS data and S-wave arrivals (Sg, SmS and Sn). a, OBS18 and **b**, OBS37. Top: Receiver gathered seismic data after band-pass filtering plotted at a reduced travel time using a reduction velocity of 4.0 km/s. T is the travel time and X is the offset. To simultaneously show the P- and S-wave arrivals, we shift the data by 2 s for display purpose. Middle: Receiver gathered seismic data with the travel time picks superimposed. Sg: Orange, SmS: Cyan, Sn: Blue. Bottom: Comparisons of the picked travel times and modelled travel times (black dots). The effects of bathymetry variation are removed in all plots by subtracting the vertical propagation time in water beneath each source, assuming a water velocity of 1.5 km/s.

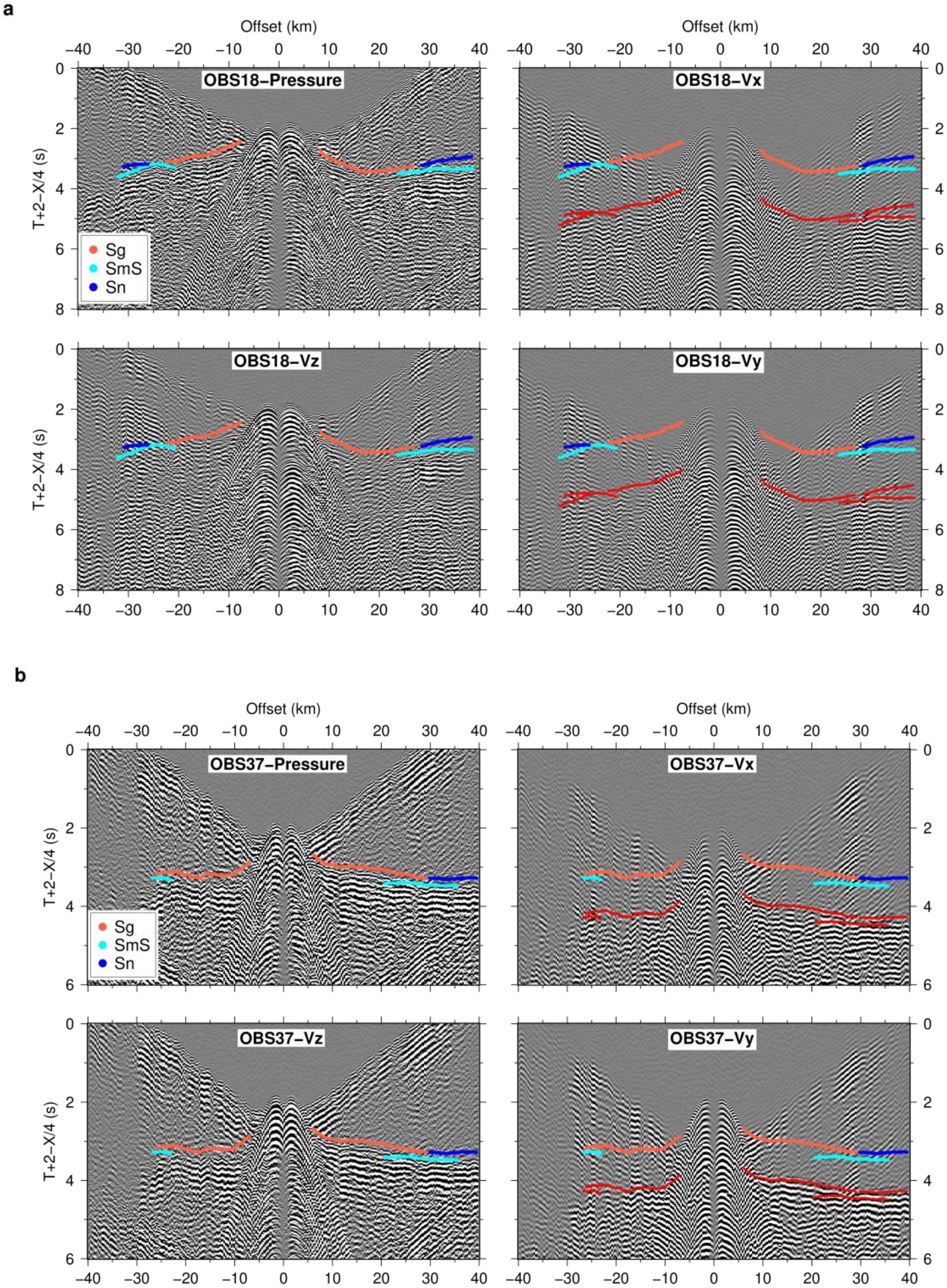

Supplementary Fig. 3. Multi-component OBS data comparison. Comparisons of the four components seismic data recorded by **a**, OBS18 and **b**, OBS37. T is the travel time and X is the offset. The seismic data after band-pass filtering are plotted at a reduced travel time using a reduction velocity of 4.0 km/s. To simultaneously show the P- and S-wave arrivals, we shift the data by 2 s. The effects of bathymetry variation are removed in all plots by subtracting the vertical propagation time in water beneath each source, assuming a water velocity of 1.5 km/s. The coloured curves show the travel times of S-wave arrivals picked on the pressured component recorded by hydrophones. At the same offset-time ranges as the picked arrivals, the S-wave arrivals are identified on the vertical component (Vz) but are not observed on the two horizontal components (Vx and Vy) of geophones, suggesting that they have travelled as a P-wave in the sediments, and the igneous crust-sediment interface was the P-to-S conversion interface. The S-waves arrivals that have travelled as S-waves in the sediments are delayed by 1.0-1.6 s due to very low S-wave velocities in the sediments (red curves).

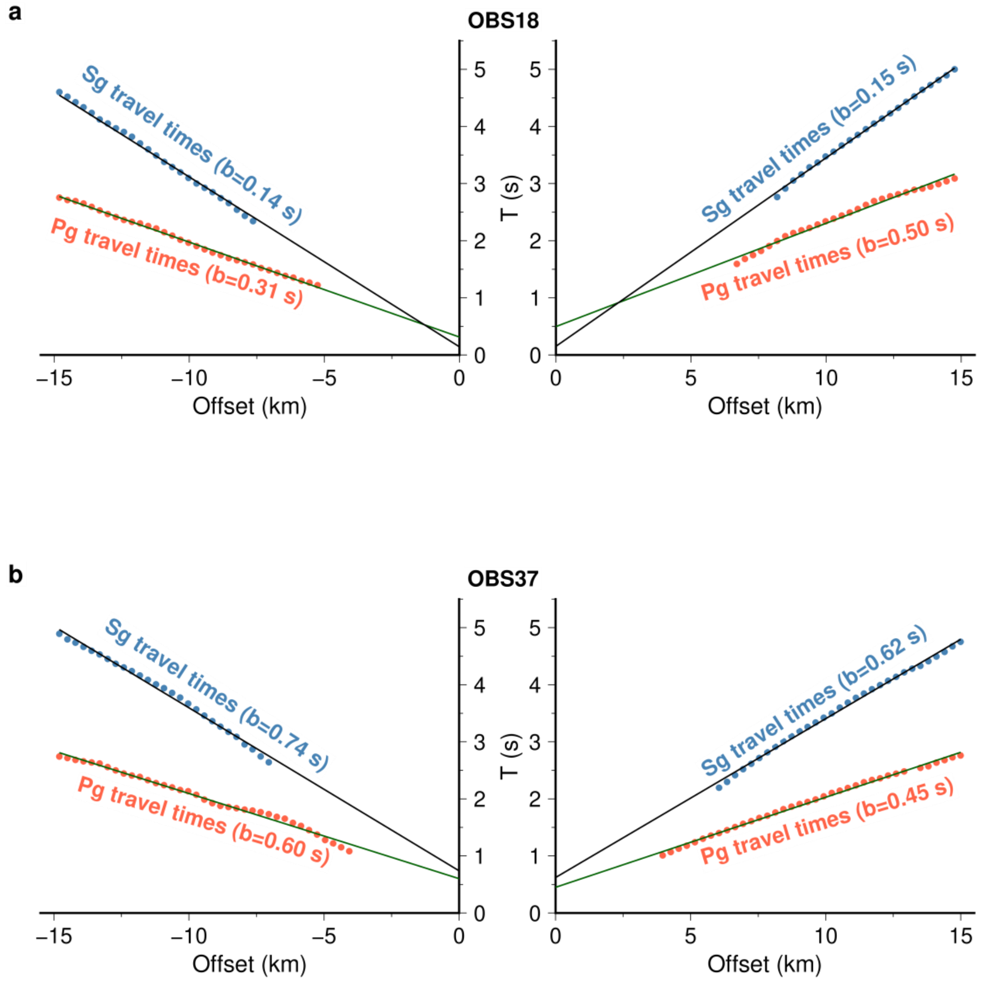

Supplementary Fig. 4. Linear regression analysis. Linear regression analysis of the travel times of Pg and Sg arrivals within ±15 km offset. **a**, for OBS18 and **b**, for OBS37. The pressure seismic data recorded by these two OBSs are shown in Supplementary Fig. 2. T is the travel time. The effects of bathymetry variation are removed in all plots by subtracting the vertical propagation time in water beneath each source, assuming a water velocity of 1.5 km/s. The picked travel times are fit using a linear regression approach for positive and negative offsets, respectively. The intercept time (**b**) from obtained linear regression analysis represents the propagation time in the sedimentary layer. The difference between the intercepts of Pg and Sg travel times are smaller than 0.35 s.

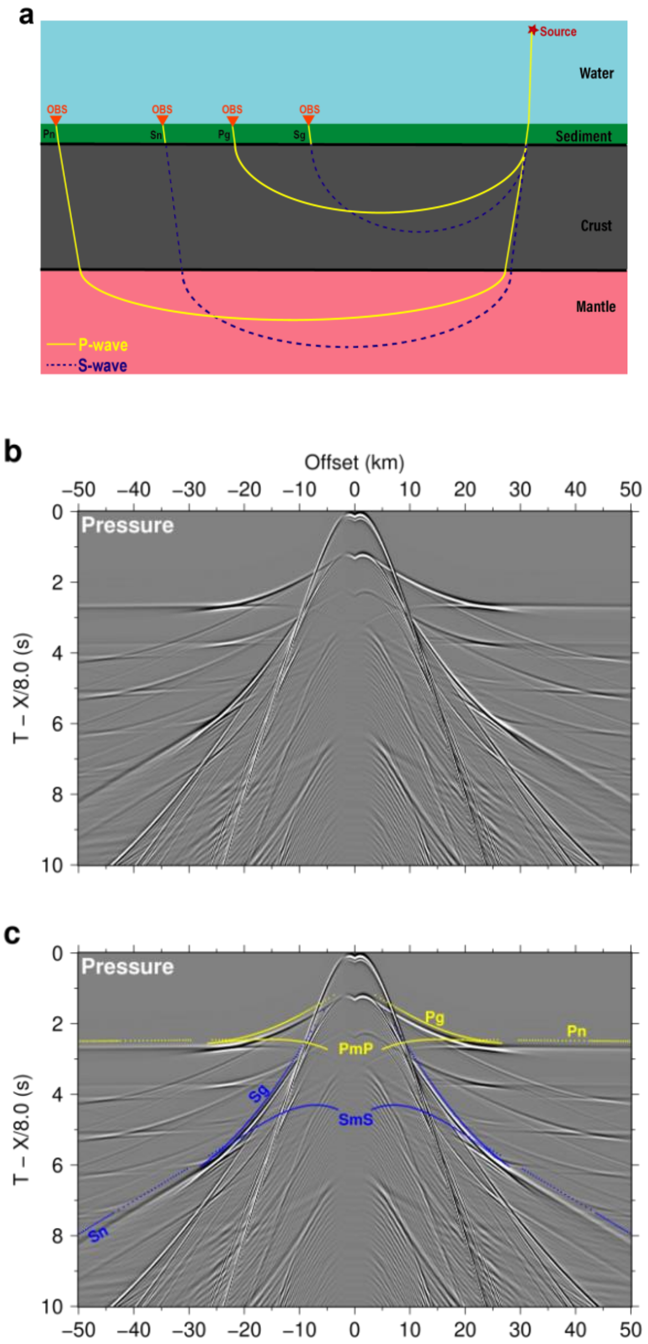

Supplementary Fig. 5. Synthetic seismogram modelling. a, Schematic diagram showing the ray paths of P-wave arrivals (Pg & Pn) and S-wave arrivals (Sg & Sn) with P-to-S and S-to-P conversions at the sediment-basement interface. **b**, Pressure data computed using a finite-difference waveform modelling algorithm[1] for the velocity model shown in **a**. The thicknesses of the water column, sediment, crust and mantle in the layered model are 5 km, 1 km, 6 km and 18 km, respectively. The P-wave velocities of the water and sediment are 1.5 km/s and 1.86 km/s, respectively. The P-wave of the crust increases from 4.5 km/s to 7.2 km/s, and that of the mantle increases from 7.9 km/s to 8.1 km/s. The S-wave velocity of sediment is 0.6 km/s. The Vp/Vs ratio of crust and mantle is 1.74. **c**, Same as **b** with the travel times of different arrivals overlapping on the seismogram. The labelled arrival phases are identified by matching their travel times calculated using the shortest path method[2].

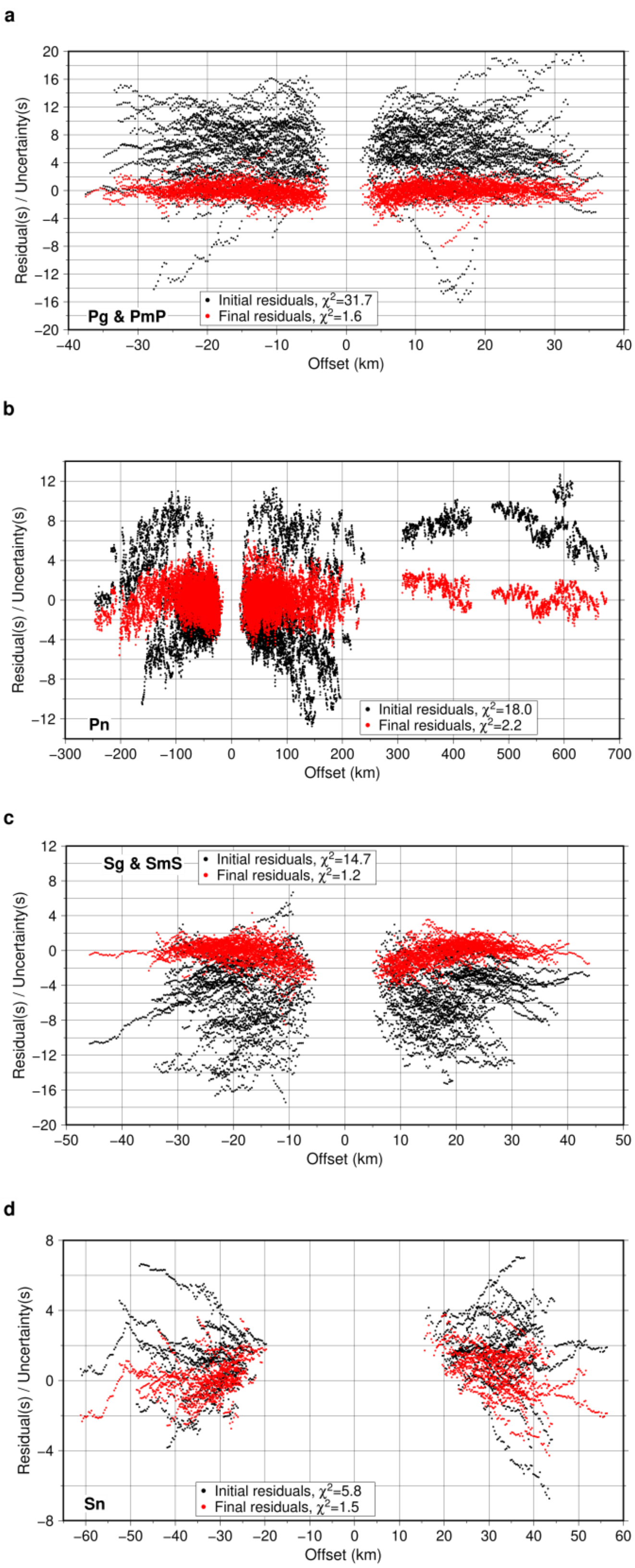

Supplementary Fig. 6. Normalised travel time residuals. Normalised travel time residuals as a function of the source-receiver offsets in the initial model (black dots) and the final tomographic model (red dots) **a,** Pg and PmP arrivals; **b,** Pn arrivals; **c,** Sg and SmS arrivals; **d,** Sn arrivals.

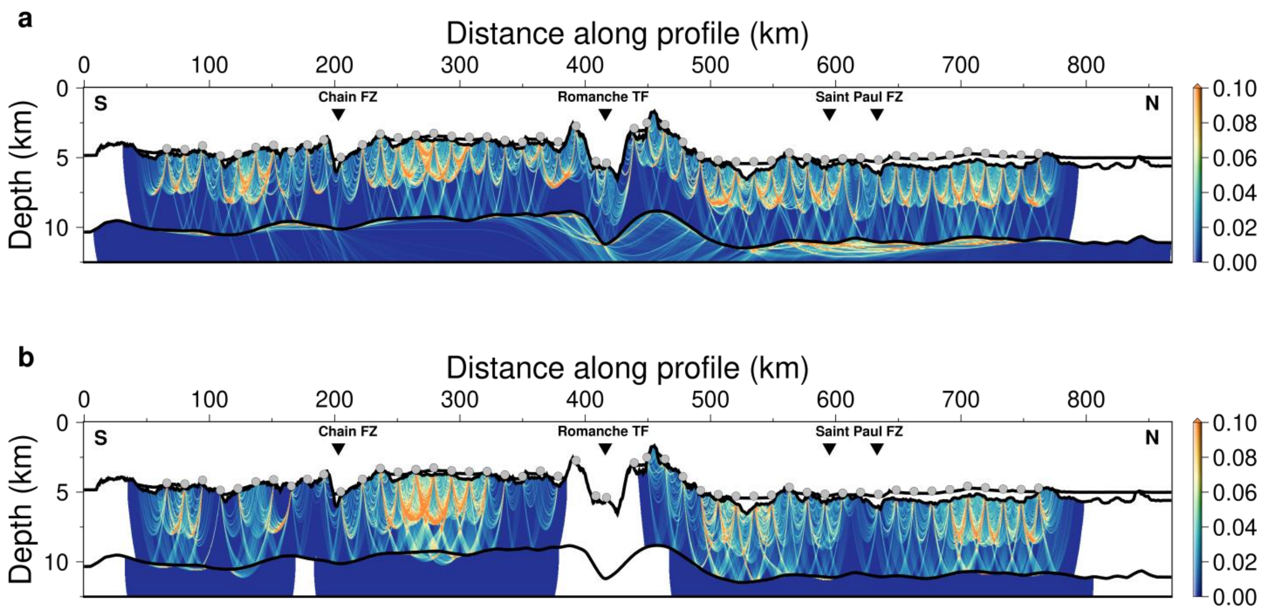

Supplementary Fig. 7. Ray coverage. The ray coverage density for **a**, P-wave arrivals and **b**, S-wave arrivals. The colour bars on the right indicate the ray density. The grey circles represent the OBSs.

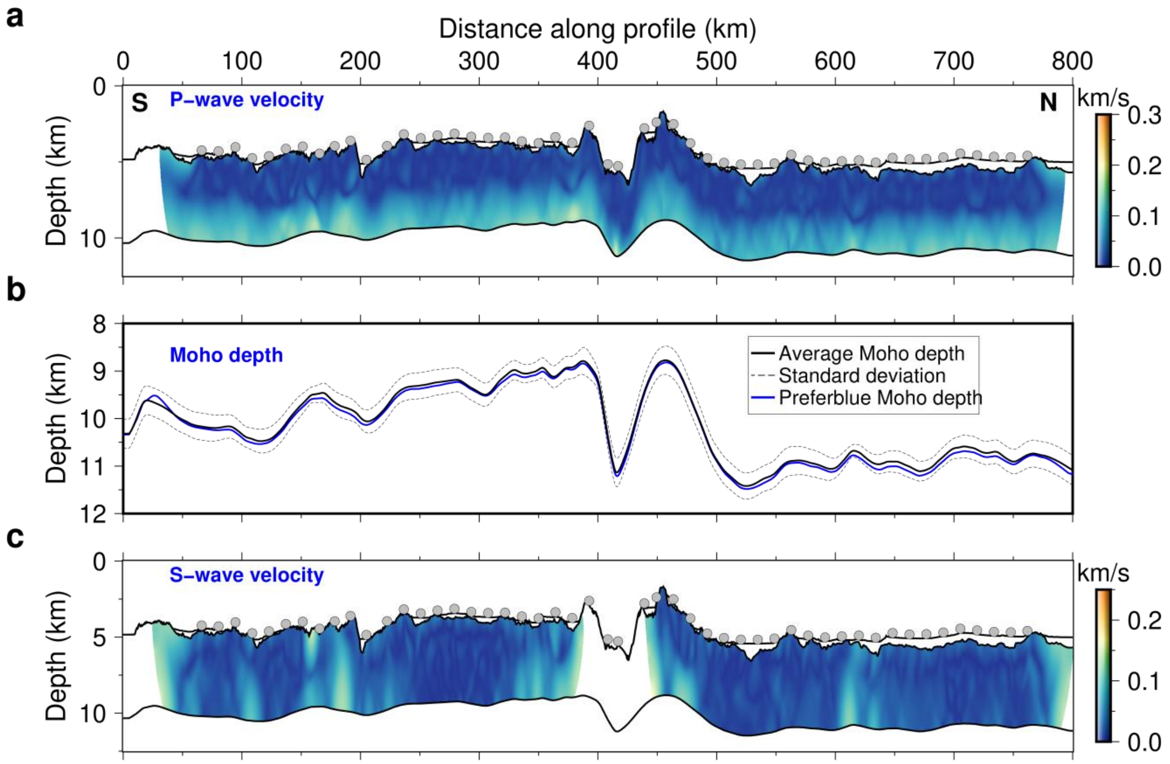

Supplementary Fig. 8. Model uncertainty from the Monte-Carlo analysis. a, Uncertainty in the crustal Vp. **b**, Uncertainty in the Moho depth. The maximum standard deviation of the Moho depth is ~400 m. **c**, Uncertainty in the crustal Vs. The colour bars on the right indicate the uncertainty in the velocity. The grey circles represent the OBSs.

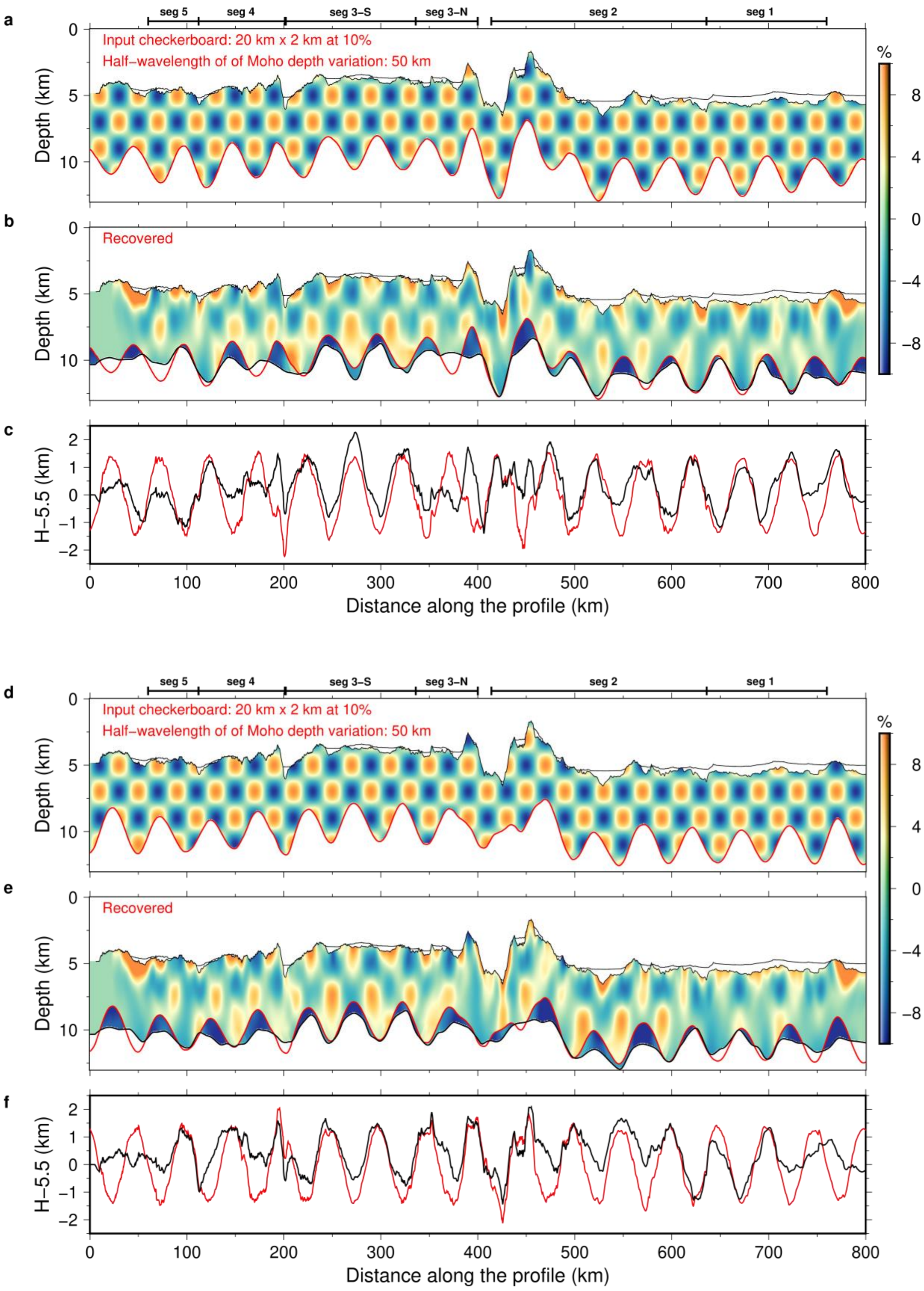

Supplementary Fig. 9. Checkerboard tests for crustal P-wave velocity. a,d, The input checkerboard pattern is 20 km × 2 km with a maximum velocity perturbation of 10%. The velocity perturbations have different polarity in **a** and **d,** so do the perturbations in Moho depth. The perturbation added to Moho depth has a half-wavelength of 50 km. **b,e**, The recovered velocity anomalies using the same tomography method as that for the picked Pg and PmP travel times. The red and black curves represent the Moho in the checkerboard model and the recovered Moho after tomography, respectively. **c,f**, Comparisons of the real crustal thickness in the checkerboard model (in red) and the recovered crustal thickness after tomography (in black).

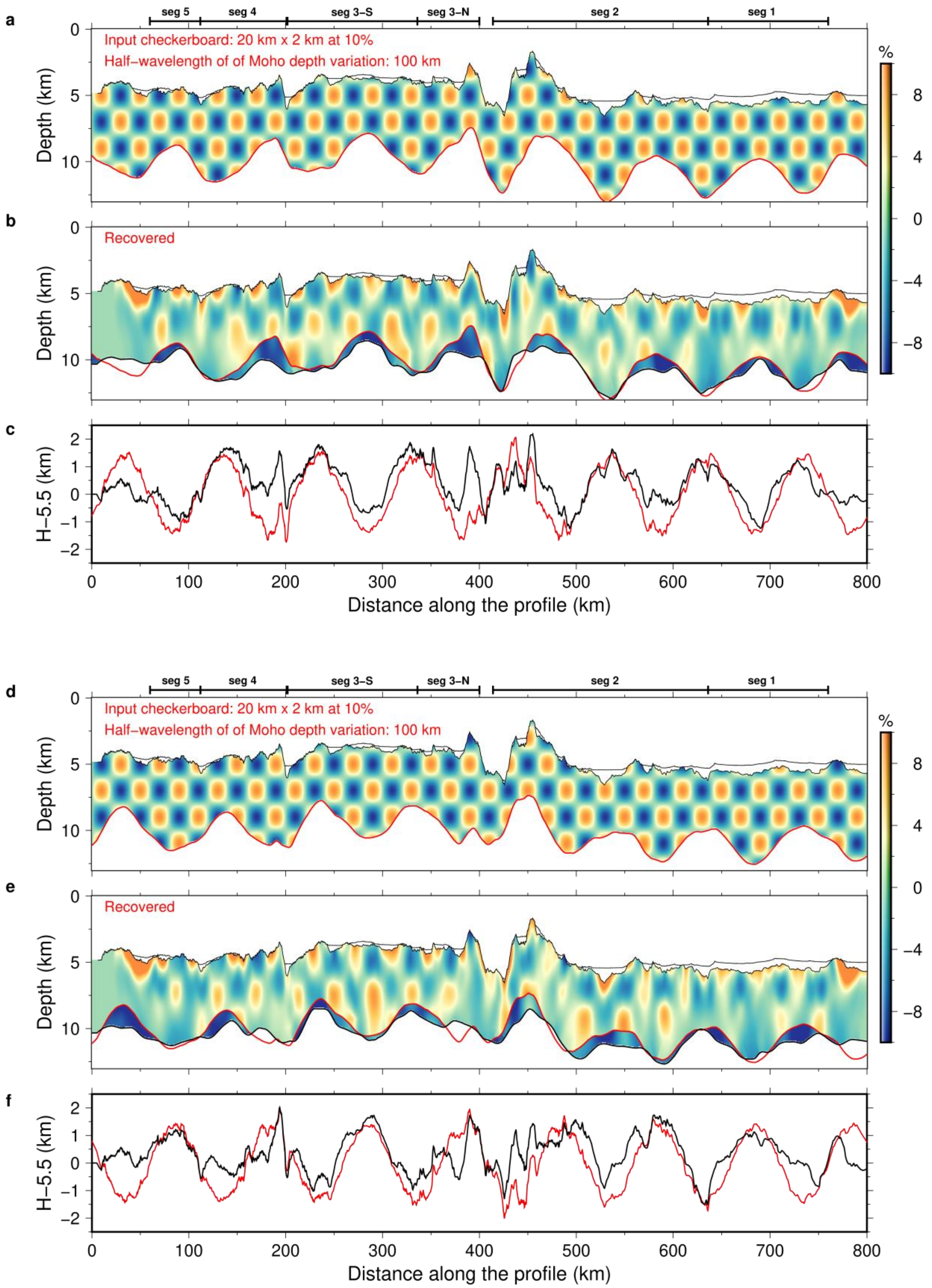

Supplementary Fig. 10. Checkerboard tests for crustal P-wave velocity. a,d, The input checkerboard pattern is 20 km × 2 km with a maximum velocity perturbation of 10%. The velocity perturbations have different polarity in **a** and **d,** so do the perturbations in Moho depth. The perturbation added to Moho depth has a half-wavelength of 100 km. **b,e**, The recovered velocity anomalies using the same tomography method as that for the picked Pg and PmP travel times. The red and black curves represent the Moho in the checkerboard model and the recovered Moho after tomography, respectively. **c,f**, Comparisons of the real crustal thickness in the checkerboard model (in red) and the recovered crustal thickness after tomography (in black).

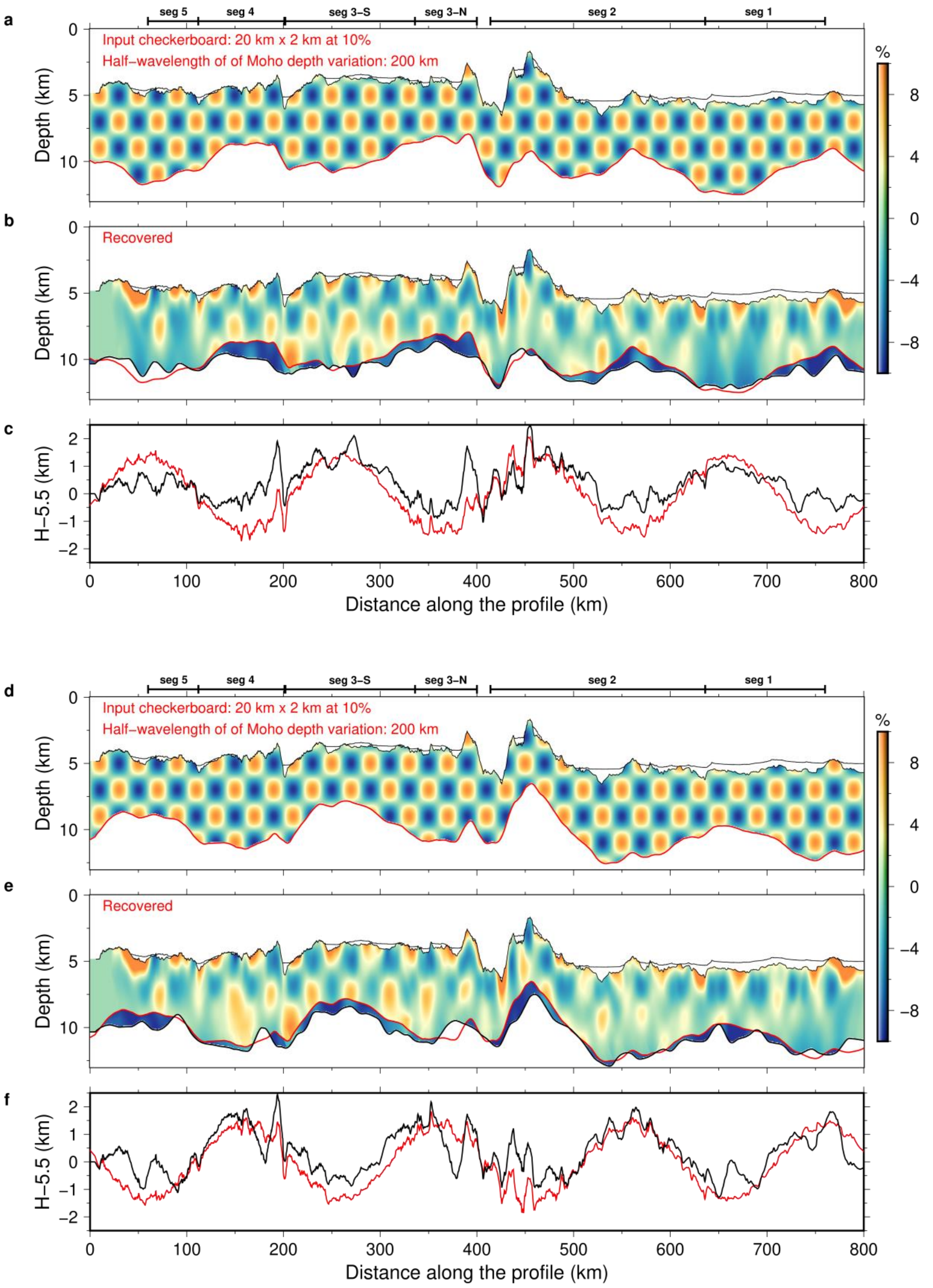

Supplementary Fig. 11. Checkerboard tests for crustal P-wave velocity. a,d, The input checkerboard pattern is 20 km × 2 km with a maximum velocity perturbation of 10%. The velocity perturbations have different polarity in **a** and **d,** so do the perturbations in Moho depth. The perturbation added to Moho depth has a half-wavelength of 200 km. **b,e**, The recovered velocity anomalies using the same tomography method as that for the picked Pg and PmP travel times. The red and black curves represent the Moho in the checkerboard model and the recovered Moho after tomography, respectively. **c,f**, Comparisons of the real crustal thickness in the checkerboard model (in red) and the recovered crustal thickness after tomography (in black).

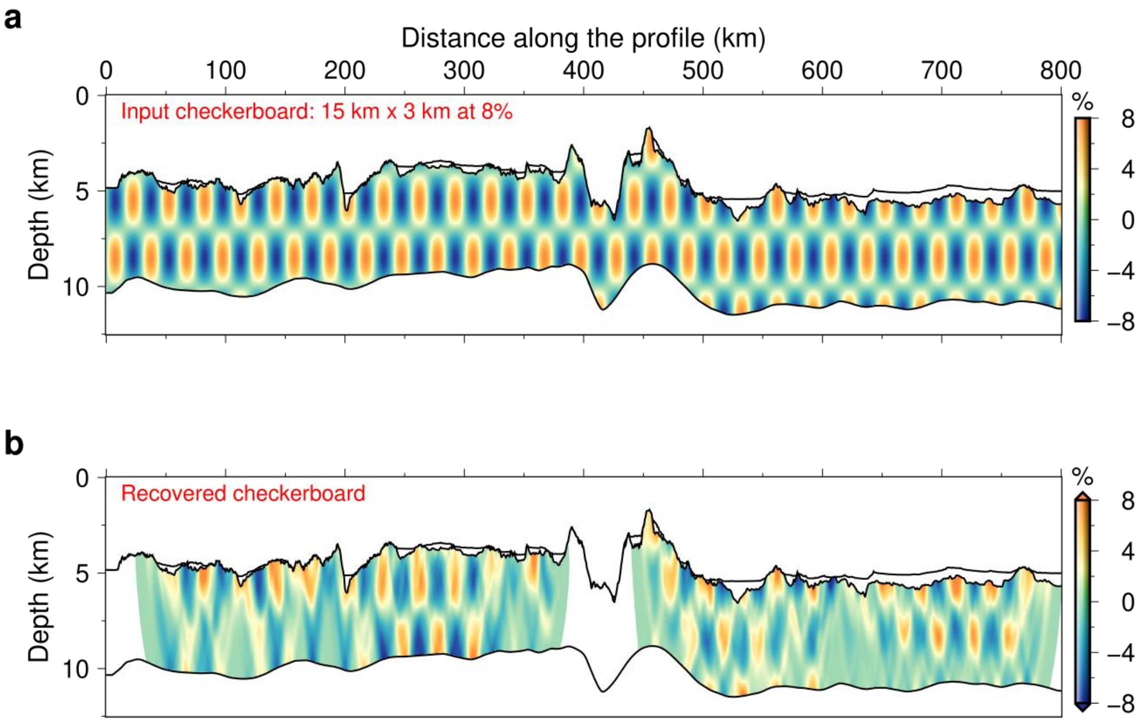

Supplementary Fig. 12. Checkerboard test for crustal Vs. a, The input checkerboard pattern with a maximum velocity perturbation of 8%. The size of the velocity anomaly is of 15 km × 3 km. **b**, The recovered velocity anomalies using the same tomography method as that for the picked Sg and SmS travel times.

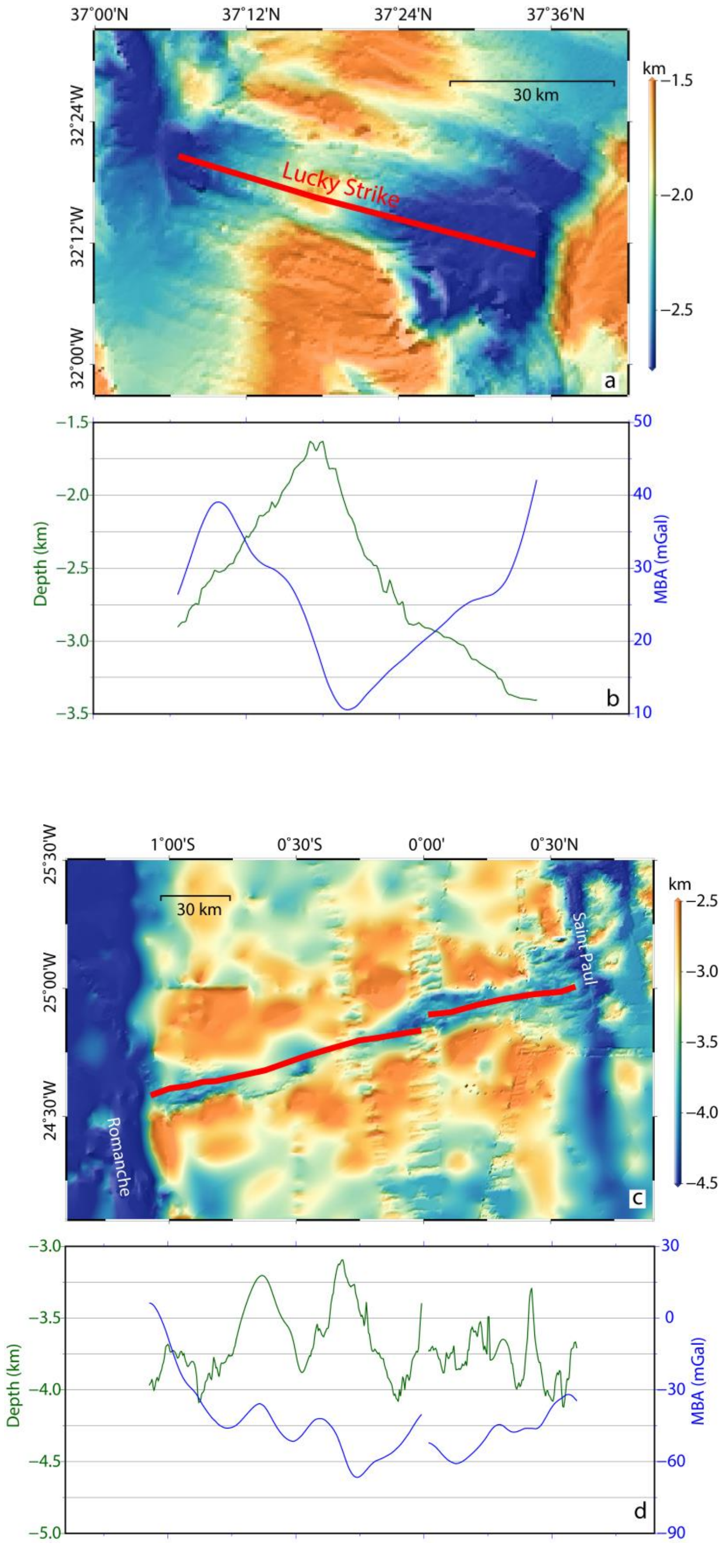

Supplementary Fig. 13. Comparison of the along-axis variations in the seafloor depth (green curves) and the mantle Bouguer anomaly (MBA; blue curves). a,b for the Lucky Strike segment and **c,d** for the segment between the St. Paul and Romanche TFs. The red curves in **a,c** represent the ridge axis where the seafloor depth and MBA are extracted and shown in **b,d**. The satellite-derived free-air gravity[3] is processed using Generic Mapping Tools[4] to obtain the MBA assuming an average crustal thickness of 5.5 km. The densities used in the calculation are 1035 kg/m^3, 2700 kg/m^3 and 3300 kg/m^3 for seawater, crust and mantle, respectively.

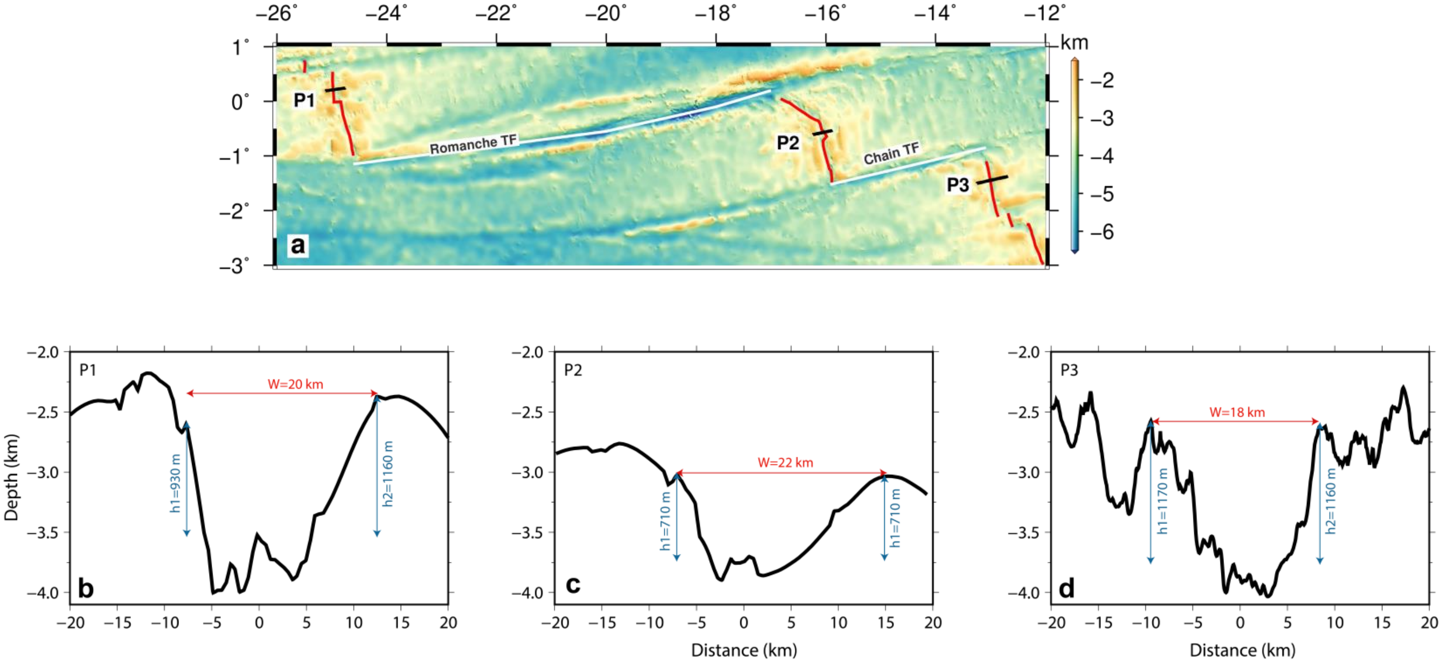

Supplementary Fig. 14. Width of the rift valley (W) and axial depth reliefs (h1 and h2) along three profiles (P1, P2 and P3). The locations of the three profiles are shown in **a.** The red curves in **a** show the ridge axis. The width of the rift valley is defined as the distance between the first steep walls on both sides of the ridge axis. The axial depth relief is defined as the depth difference between the crest of the axial valley and the valley wall.

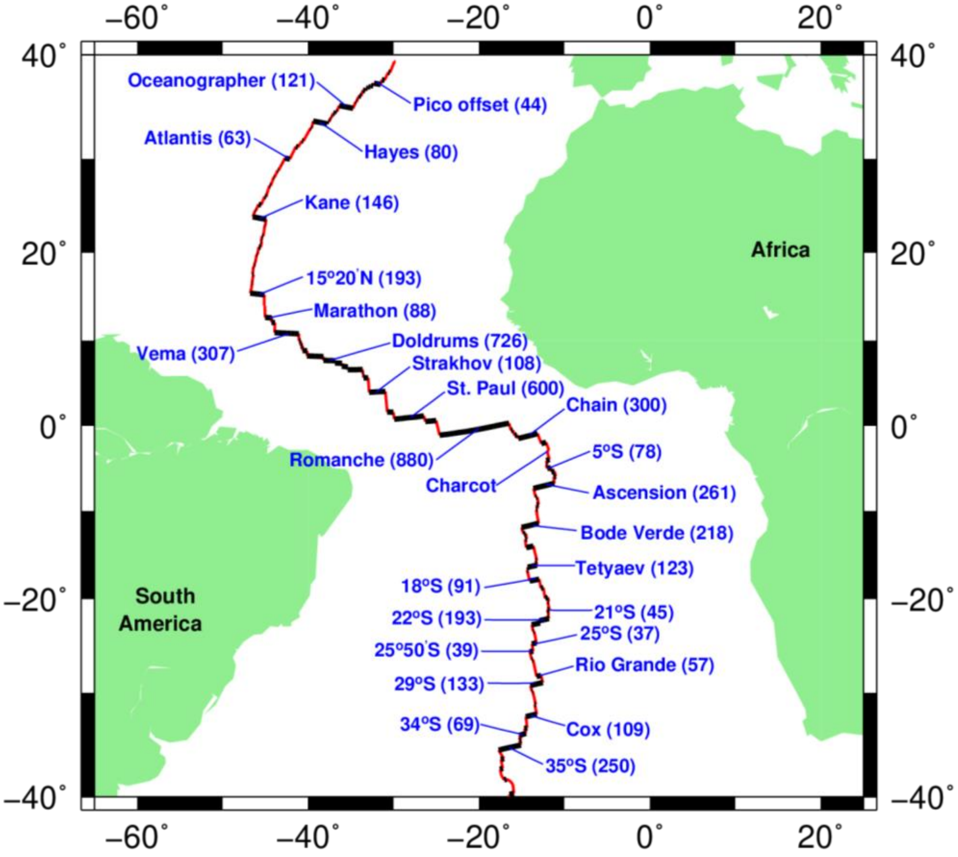

Supplementary Fig. 15. Map showing the locations and lengths of oceanic transform faults (TFs) in the Atlantic Ocean between 40°N and 40°S. The numbers in brackets are the lengths of the oceanic TFs with unit in km. Only oceanic TFs with length >30 km are plotted. For an oceanic TF composed of several intra-transform faults, the total length of the TF is labelled.

Supplementary Table 1. Interpreted average thicknesses and average vertical Vp gradients of crustal Layers 2 and 3. The vertical Vp gradient of 0.5 s^{-1} is used as the Layer 2/3 boundary[5]. The errors represent the standard deviations.

	**Thickness of Layer 2 (km)**	**Average Vp gradient of Layer 2 (s^{-1})**	**Thickness of Layer 3 (km)**	**Average Vp gradient of Layer 3 (s^{-1})**
**Segment 1**	2.1 ±0.2	0.76 ±0.12	3.3 ±0.2	0.15 ±0.04
**Segment 2**	2.2 ±0.6	0.67 ±0.14	3.4 ±0.6	0.17 ±0.07
**Segment 3-S**	1.9 ±0.7	0.66 ±0.26	3.5 ±0.7	0.17 ±0.05
**Segment 4**	2.3 ±0.6	0.80 ±0.19	3.1 ±0.6	0.15 ±0.10
**Segment 5**	2.2 ±0.3	0.73 ±0.12	3.4 ±0.3	0.13 ±0.04

Supplementary Table 2. Crustal thickness at the centre and ends of segments along the slow-spreading Mid-Atlantic ridge and fast-spreading East Pacific Rise. The crustal thickness data from the Atlantic Ocean are selected following two criteria: (1) systematically along-axis crustal thinning is observed within the second-order ridge segment and (2) the crustal thicknesses at segment centre and at least one segment end are measured.

Ocean	Location of ridge (reference)	Name of ridge or seismic profile	Spreading half rate (mm/yr)	Age of crust (Myr)	Crustal thickness at segment centre (km)	Crustal thickness at segment end(s) (km)
North Atlantic Ocean	52 ^{o}N	Profile 10617[6]	12.7	~5.1	8	5.0
	33-35°N	OH-1[7]	~11	0	8.2	5.0 / 5.0
		OH-2[7]		0	6.9	3.3 / 4.4
		OH-3[7]		0	6.6	2.5 / 4.2
	35°N	OH-1[8]		~2	8.1	3.8 /4.6
		OH-2[8]		~2	7.0	3.8
	35°N	OH-1[9]		~2	9.0	4.5 / 6.0
		OH-1[9]		~5	6.0	3.2 / 3.5
	21.5^{o}N	TAMMAR[10]	~13	0	8.0	4.0 / 5.5
	14.5^{o}N	Segment 4[11]	~23	~70	7.6	4.2 / 5.3
South Atlantic Ocean	33°S	33°S segment[12]	~18	0	7.8	3.5
	8-9°S	segment A2[13]	~16	~0	10	6.0 / 7.0
	5°S	Profile 10[14]	~16	~0.8	8.5	2.8 / 3.5
East Pacific Ocean	15°N	Line 1<br>Line 2[15]	~46.5	0.8-2.0	6.0	5.4
	14.3^{o}N	CLASSIC deployment 1[16]	~55	0.6-0.9	5.8	5.6
	8°15'–10°5'N	Southern segment on Cocos Plate[17]	~55	0.3	6.8	5.3
		Northern segment on Cocos Plate[17]		0.3	7.3	~6.0
	3.5^{o}–5°N	G3 profile[18]	~70	0.58-2	6.6	5.0
		Q1 profile[18]		0.28-1.42	6.0	5.0

Supplementary Table 3. Maximum crustal thickness variation between centre and ends of second-order ridge segments and the length of the corresponding first-order ridge segment in the Atlantic Ocean. The locations of the transform faults are shown in Supplementary Fig. 15.

Transform faults bound the first-order ridge segment	Length of the first-order segment (km)	Maximum crustal thickness variation within the segment (km)	Age of the measured crust (Myr)	Seismic or Gravity data
Pico Offset—Oceanographer TF	~360	4.0[19]	0	Gravity data
Oceanographer TF—Hayes TF	~220	4.1[7]	0	Seismic data
		4.3[8]	~2	
		2.8[9]	~5	
Atlantis TF—Kane TF	~800	3.5[20]	0	Gravity data
Kane TF—15°20'N TF	~930	4.0[10]	0	Seismic data
15°20'N TF—Marathon TF	~270	3.4[11]	~65	Seismic data
Charcot TF—5°S TF	~230	5.7[14]	~0.8	Seismic data
Ascension TF—Bode Verde TF	~450	4.0[13]	0	Seismic data
Cox TF— 34°S TF	~190	4.3[12]	0	Seismic data

Supplementary Table 4. Lengths of oceanic transform faults (TFs) and non-transform offsets (NTOs) in the Atlantic Ocean and the crustal thinning towards these TFs and NTOs. Only the crustal thickness constrained by active-source seismic data is considered.

TF or NTO	Length of TF or NTO (km)	Location of the measured crust	Age of crust (Myr)	Crustal thickness at segment centre (km)	Crustal thickness at TF or NTO (km)
Charlie-Gibbs TF	~350	South of TF	~5.1	8	5.0[6]
Oceanographer TF	~121	South of TF	0	8.2	5.0[7]
			~2	8.1	4.6[8]
			~2	9.0	4.5[9]
			~5	6.0	3.5[9]
NTO-1 at 34.5°N	~35	South of NTO-1	0	6.9	3.3[7]
			~2	7.0	3.8[8]
		North of NTO-1	0	8.2	5.0[7]
			~2	8.1	3.8[8]
			~2	9.0	6.0[9]
			~5	6.0	3.2[9]
NTO-2 at 34°N	~35	South of NTO-2	0	6.6	4.2[7]
		North of NTO-2	0	6.9	4.4[7]
NTO-3 at 33.5°N	~15	North of NTO-3	0	6.6	2.5[7]
Marathon TF	~88	North of TF	~70	7.6	5.3[11]
Ascension TF	~261	South of TF	0	10	6.0[13]
5°S TF	~70	North of TF	~0.8	8.5	2.8[14]

References

1. Levander, A. R. Fourth‐order finite‐difference P-SV seismograms. *Geophysics* **53**, 1425-1436 (1988).

2. Moser, T. J. Shortest path calculation of seismic rays. *Geophysics* **56**, 59-67 (1991).

3. Sandwell, D. T., Müller, R. D., Smith, W. H. F., Garcia, E. & Francis, R. New global marine gravity model from CryoSat-2 and Jason-1 reveals buried tectonic structure. *Science* **346**, 65-67 (2014).

4. Wessel, P. & Smith, W. H. F. Free software helps map and display data. *Eos, Transactions American Geophysical Union* **72**, 441-446 (1991).

5. Vaddineni, V. A., Singh, S. C., Grevemeyer, I., Audhkhasi, P. & Papenberg, C. Evolution of the Crustal and Upper Mantle Seismic Structure From 0–27 Ma in the Equatorial Atlantic Ocean at 2° 43′ S. *J. Geophys. Res.* **126**, e2020JB021390 (2021).

6. Whitmarsh, R. B. & Calvert, A. J. Crustal structure of Atlantic fracture zones — I. The Charlie-Gibbs Fracture Zone. *Geophys. J. Int.* **85**, 107-138 (1986).

7. Hooft, E. E. E., Detrick, R. S., Toomey, D. R., Collins, J. A. & Lin, J. Crustal thickness and structure along three contrasting spreading segments of the Mid-Atlantic Ridge, 33.5°–35°N. *J. Geophys. Res.* **105**, 8205-8226 (2000).

8. Canales, J. P., Detrick, R. S., Lin, J., Collins, J. A. & Toomey, D. R. Crustal and upper mantle seismic structure beneath the rift mountains and across a nontransform offset at the Mid-Atlantic Ridge (35°N). *J. Geophys. Res.* **105**, 2699-2719 (2000).

9. Sinha, M. C. & Louden, K. E. The Oceanographer fracture zone — I. Crustal structure from seismic refraction studies. *Geophys. J. Int.* **75**, 713-736 (1983).

10. Dannowski, A., et al. Crustal structure of the propagating TAMMAR ridge segment on the Mid-Atlantic Ridge, 21.5°N. *Geochem. Geophys. Geosyst.* **12**, Q07012 (2011).

11. Davy, R. G., Collier, J. S., Henstock, T. J. & The VoiLA Consortium. Wide-Angle Seismic Imaging of Two Modes of Crustal Accretion in Mature Atlantic Ocean Crust. *J. Geophys. Res.* **125**, e2019JB019100 (2020).

12. Tolstoy, M., Harding, A. J. & Orcutt, J. A. Crustal Thickness on the Mid-Atlantic Ridge: Bull's-Eye Gravity Anomalies and Focused Accretion. *Science* **262**, 726-729 (1993).

13. Minshull, T. A., Bruguier, N. J. & Brozena, J. M. Ridge-plume interactions or mantle heterogeneity near Ascension Island? *Geology* **26**, 115-118 (1998).

14. Planert, L., Flueh, E. R. & Reston, T. J. Along- and across-axis variations in crustal thickness and structure at the Mid-Atlantic Ridge at 5°S obtained from wide-angle seismic tomography: Implications for ridge segmentation. *J. Geophys. Res.* **114**, B09102 (2009).

15. Tréhu, A. M. & Purdy, G. M. Crustal structure in the Orozco Transform Zone. *J. Geophys. Res.* **89**, 1834-1842 (1984).

16. Van Avendonk, H. J. A., Harding, A. J., Orcutt, J. A. & McClain, J. S. A two-dimensional tomographic study of the Clipperton transform fault. *J. Geophys. Res.* **103**, 17885-17899 (1998).

17. Canales, J. P., Detrick, R. S., Toomey, D. R. & Wilcock, W. S. D. Segment-scale variations in the crustal structure of 150–300 kyr old fast spreading oceanic crust (East Pacific Rise, 8° 15′ N–10° 5′ N) from wide-angle seismic refraction profiles. *Geophys. J. Int.* **152**, 766-794 (2003).

18. Roland, E., Lizarralde, D., McGuire, J. J. & Collins, J. A. Seismic velocity constraints on the material properties that control earthquake behavior at the Quebrada-Discovery-Gofar transform faults, East Pacific Rise. *J. Geophys. Res.* **117**, B11102 (2012).

19. Detrick, R. S., Needham, H. D. & Renard, V. Gravity anomalies and crustal thickness variations along the Mid-Atlantic Ridge between 33 °N and 40 °N. *J. Geophys. Res.* **100**, 3767-3787 (1995).

20. Lin, J., Purdy, G. M., Schouten, H., Sempere, J. C. & Zervas, C. Evidence from gravity data for focused magmatic accretion along the Mid-Atlantic Ridge. *Nature* **344**, 627-632 (1990).
